# Identification and Analysis of Genetic Variations in Pri-MiRNAs Expressed Specifically or at a High Level in Sheep Skeletal Muscle

**DOI:** 10.1371/journal.pone.0117327

**Published:** 2015-02-20

**Authors:** Wei Zhang, Limin Wang, Ping Zhou, Guangchao Song, Min Shen, Shangquan Gan, Guoqing Shi

**Affiliations:** 1 College of Animal Science and Technology, Shihezi University, Shihezi, 832003, China; 2 Institute of Animal Husbandry and Veterinary Medicine, Xinjiang Academy of Agricultural and Reclamation Sciences, Shihezi 832000, China; University of Bonn, GERMANY

## Abstract

MicroRNAs (miRNAs) are key regulators in miRNA-mediated gene regulatory networks and play important roles in many biological processes, such as growth and development of mammals. In this study, we used microarrays to detect 261 miRNAs that are expressed in sheep skeletal muscle. We found 22 miRNAs that showed high levels of expression and equated to 89% of the total miRNA. Genetic variations in these 22 pri-miRNAs were further investigated using polymerase chain reaction-single strand conformation polymorphism (PCR-SSCP) and sequencing. A total of 49 genetic variations, which included 41 single nucleotide polymorphisms (SNPs) and 8 deletions/insertions, were identified in four sheep breeds. Three variations were further researched in a larger sample set, including five sheep breeds with different meat production performances. We found that the genotype and allele frequencies of the CCC deletion/insertion in pri-miR-133a were significantly related to the sheep meat production trait. Finally, cell assays and quantitative reverse transcription PCR (qRT-PCR) were employed to investigate the effect of pri-miRNA genetic variation on the miRNA biogenesis process. The results confirmed that genetic variations can influence miRNA biogenesis and increase or decrease the levels of mature miRNAs, in accordance with the energy and stability change of hair-pin secondary structures. Our findings will help to further the understanding of the functions of genetic variations in sheep pri-miRNAs in skeletal muscle growth and development.

## Introduction

MicroRNAs (miRNAs) are a class of endogenous, non-coding RNAs of approximately 22 nucleotides (nt) in length. They play an important role in post-transcriptional regulation [1]. MicroRNA biogenesis requires the participation of many proteins in two steps in the nucleus and cytoplasm. In the nucleus, the majority of miRNA genes are transcribed by RNA polymerase II (the minority by RNA polymerase III) and generate approximately one kilobase (kb) long primary transcripts called pri-miRNAs. These contain one or more hair-pin structure regions of approximately 80 nt. An RNase III endonuclease, named Drosha, binds to pri-miRNAs and releases the miRNA precursor, pre-miRNAs [2]. After export to the cytoplasm by Exportin 5 [3], pre-miRNAs are further processed by another RNase III endonuclease, named Dicer, into mature miRNA duplexes. One strand of the mature miRNA duplex is assembled by the Argonaut protein within the RNA-induced silencing complex (RISC). This complex regulates the gene expression of its target by translational repression or mRNA cleavage [4].

There is increasing evidence that miRNAs are key regulators of gene expression and play crucial roles in diverse biological processes [5, 6]. Thirty percent of human genes have been shown to be miRNA targets [7]. A change in miRNA transcript or maturation processing, caused by genetic variation, will have a significant influence on the expression of various protein-coding genes and may affect phenotype or disease susceptibility [8]. Although miRNA genes are conserved in the general population because of selection pressure, evidence suggests that extensive genetic variations exist and may have important biological functions [9, 10]. For example, the single nucleotide polymorphisms (SNPs) found in miR-146a (C>G, rs2910164), miR-149 (T>C, rs2292832) and miR-196a2 (T>C, rs11614913) are associated with gastric cancer susceptibility in the Greek population [11]. A miR-96 (+57 T>C) mutation may reduce the stability of the pre-miRNA hair-pin structure, down-regulate the miR-96 level and contribute to non-syndromic inherited hearing loss in humans [12]. Two miR-185 SNPs (C>T, rs2008591 and A>G, rs887205) have shown an inverse association with breast cancer risk amongst African Americans [13].

Genetic variations in pri-miRNAs and their disease associations have been extensively studied in humans but there has been a lack of focus on functions in other biological processes, such as myogenesis and proliferation. Recent research has demonstrated that miRNAs play important roles in mammalian skeletal muscle differentiation and proliferation as they regulate the genes expressed in skeletal muscle [14–16]. Genetic variations of these miRNA genes may have significant influence over the expression of their targets and, consequently, over skeletal muscle growth and development. However, there are no studies on genetic variations of miRNA in skeletal muscle of sheep.

Recent studies have confirmed that miR-133 enhances myoblast proliferation by repression of the serum response factor (SRF) [16]. It also determines whether skeletal muscle satellite cells differentiate into the myogenic lineage or brown adipocytes, by targeting the 3′ untranslated region (UTR) of *Prdm16* [17]. In contrast, miR-1 promotes myogenesis by targeting histone deacetylase 4 (*HDAC4*) [16]. Another miRNA, miR-206, is able to repress myoblast fusion by targeting the connexin 43 (*Cx43*) gap junction channels without altering the *Cx43* mRNA level [18]. As myogenesis and proliferation are sophisticated biological pathways, we speculated that there are many more miRNAs included in these processes but investigations are rare in sheep.

The objectives of this study were 1) to use a miRNA microarray to obtain miRNA transcriptome profiles during skeletal muscle development in sheep; 2) to identify the genetic variations of miRNA genes that are expressed specifically or at a high level in sheep skeletal muscle and to investigate the effect of these variations on miRNA biogenesis.

This research may help to recognize the genetic variations in sheep miRNA genes. Together with future studies on miRNA target genes, this may lead to a greater understanding of the functions of miRNAs in sheep skeletal muscle growth.

## Results

### miRNA expression in sheep skeletal muscle

To investigate miRNAs that are expressed in sheep skeletal muscle, a miRNA microarray (Exiqon microRNA Array V.9.2, Exiqon, Denmark) with 364 probes complementary to the 253 hsa-miRNAs and 111 bta-miRNAs found in mammalian skeletal muscle was used (S1 Table) [19, 20, 21].

The array dataset was already submitted to ArrayExpress, and the accession number is E-MTAB-3096 (http://www.ebi.ac.uk/arrayexpress/). According to the signal detection criteria, 261 miRNAs were detected in sheep skeletal muscle but their expression levels showed significant differences (S2 Table). Some miRNAs, especially those expressed specifically in mammalian skeletal muscle, such as miR-1, miR-133 and miR-206, had extremely high expression levels. However, the expression levels of some miRNAs, such as miR-190, miR-147, miR-433, etc., were very low. We further analyzed these 261 detected miRNAs, and found that there were 22 miRNAs whose expression levels were ten thousand-fold greater than that of the others. We hypothesized that these 22 miRNAs with higher expression may play an important role in sheep skeletal muscle growth and development. Therefore, we focused on their genetic variations and relative functions.

**Fig 1:**
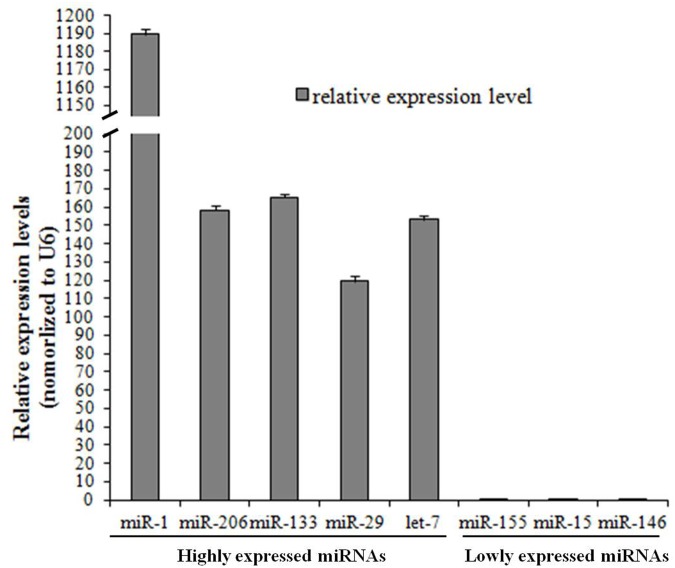
Validation of the microarray results using qRT-PCR. Five highly expressed miRNAs and three lowly expressed miRNAs were chosen according to the microarray dataset to detect their expression levels in sheep skeletal muscle. The graph was generated using MS Excel. Bar graphs show the mean ± SEM.

To validate the accuracy of the microarray data, five highly expressed miRNAs (miR-1, miR-206, miR-133, miR-29 and let-7) and three lowly expressed miRNAs (miR-155, miR-15 and miR-146) were chosen, and their expression in the biceps femoris muscle of adult (12-month-old) Altay sheep were detected using qRT-PCR. The results showed that the expression levels of these eight miRNAs conformed to the microarray data; thus, the microarray data were reliable (Fig. 1).

### The genetic variations of pri-miRNAs expressed in sheep skeletal muscle

The genetic variations in the 22 miRNA genes were investigated with PCR-SSCP and sequencing. A total of 41 SNPs in 16 pri-miRNAs were identified in different sheep breeds (Table 1). All are novel and are not found in miRSNP (release 2.0, July 2013) or dbSNP (Build 141, May 2014). In addition, eight deletions/insertions were detected in six pri-miRNAs for the first time (Table 2). When Blat of UCSC Genome Browser (http://genome.ucsc.edu/) was applied, the pri-miRNA sequences were mapped to the sheep genome (ISGC Oar_v3.1/oviAri3, August 2012) and the genetic variations were located on chromosomes.

**Table 1:** SNPs in pri-miRNAs that were expressed specifically, or at a high level, in skeletal muscle of sheep.

miRNA	Chromosome	Genome Context^b^	Alleles^c^	Chromosome Position^d^	SNP Location^e^
miR-133a	13(-)^a^	intergenic	A>G	g.54047491	+273
miR-133b	20(+)	intergenic	C>T	g.24358041	+80
miR-206	20(+)	intergenic	T>C	g.24353711	-125
			C>A	g.24353995	+160
			C>T	g.34354040	+205
miR-99a	1(-)	intergenic	C>T	g.138829167	-51
			T>G	g.138828887	+230
			G>A	g.138828752	+365
miR-29a	4(-)	exonic	T>G	g.94625903	-85
			G>A	g.94625852	-34
			G>A	g.94625822	-4
miR-199a	12(-)	intergenic	T>C	g.37776122	+17
			T>C	g.37775950	+189
miR-378–2	4(+)	intronic	G>T	g.10768604	+101
miR-27b	2(-)	intronic	T>C	g.31139368	-261
			G>C	g.31138951	+156
			G>C	g.31138918	+190
			T>A	g.31138892	+218
miR-29c	12(+)	intergenic	A>G	g.73693385	-204
			G>T	g.73693392	-197
			T>C	g.73693431	-158
miR-101–2	2(+)	exonic/intronic	T>C	g.72602944	-120
			C>T	g.72603207	+100
			T>A	g.72603236	+129
			T>C	g.72603320	+213
			G>A	g.72603357	+250
			G>A	g.72603369	+262
			A>G	g.72603378	+271
miR-140	14(+)	intronic	T>G	g.36296691	-264
			C>T	g.36296699	-256
miR-143	5(+)	intergenic	G>C	g.58323340	-240
miR-128–1	2(-)	intronic	G>A	g.174018351	+219
miR-128–2	19(+)	intronic	T>C	g.9630283	-229
			T>C	g.9630316	-196
			T>C	g.9630372	-140
Let-7a	2(-)	intergenic	T>C	g.27315093	-118
			G>A	g.27314935	+45
			T>C	g.27314879	+101
			T>C	g.27314841	+131
			C>T	g.27314816	+164
Let-7c	1(-)	exonic	T>C	g.138828046	+313

Note: ^a^ +/− in brackets indicates that the miRNA is on the positive or negative strand of the DNA.

^b^ The genomic contexts of the miRNAs were obtained from miRBase 21. The genome context of miR-99a, miR-199a, miR-143 and let-7a were from the *Ovis aries* genome, and the remainder were from the *Bos taurus* genome because their information in the *Ovis aries* genome cannot be searched.

^c^ Arrows indicate the direction of the single nucleotide polymorphism.

^d^ The position of the genetic variation in the sheep genome (ISGC Oar_v3.1/oviAri3, August 2012).

^e^ Location of the genetic variation is shown as (+) downstream or (−) upstream of the 5′ nucleotide of pre-miRNA.

**Table 2:** Novel deletions/insertions detected in pri-miRNAs that are expressed at a high level, or specifically, in skeletal muscle of sheep.

MiRNA	Chr	Deletion/insertion^b^	Chromosome Position^c^	Location^d^
miR-133a	13(-)^a^	CCC	g.540047592—g.540047594	+170
miR-99a	1(-)	ATATATATATATAC	g.138828934—g.138828947	+170
		ATATATATATATATAC	g.138828918—g.138828933	+184
miR-29a	4(-)	CTT	g.94625946—g.94625948	-127
		TAATAATAC	g.94625593—g.94625601	+218
miR-29c	12(+)	CA	g.73693768—g.73693769	+179
miR-128–1	2(-)	ATCATGC	g.174018362—g.174018368	+208
miR-let7a	2(-)	T	g.27315085	-60

Note: ^a^ +/− in brackets indicates that the miRNA is on the positive or negative strand of the DNA.

^b^ The nucleotides deleted or inserted.

^c^ The position of the genetic variation in the sheep genome (ISGC Oar_v3.1/oviAri3, August 2012).

^d^ Location of the genetic variation is shown as (+) downstream or (−) upstream of the 5′ nucleotide of pre-miRNA.

In addition, six pri-miRNAs, pri-miR-1, 499, 26a, 378–1, 486 and 22, did not show any genetic variations in sheep.

### Characterization of genetic variations in pri-miRNA sequences

**Fig 2:**
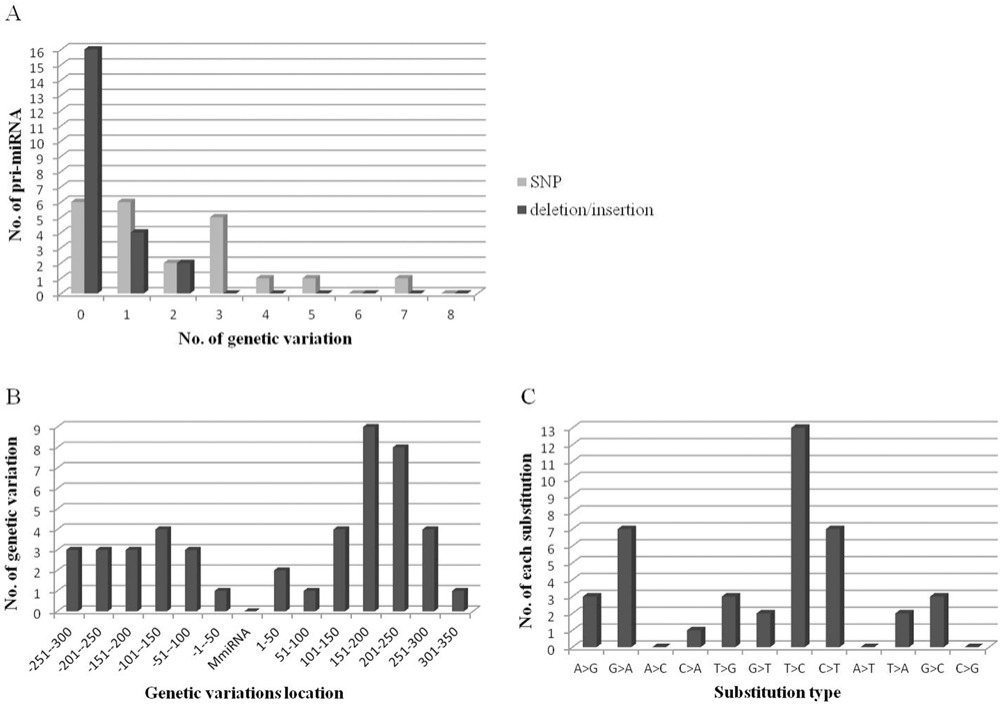
The characteristics of genetic variations in sheep pri-miRNA sequences. (A) Frequency distribution of genetic variations in pri-miRNAs. (B) The location of genetic variations in pri-miRNAs. The 5′ nucleotide of pre-miRNA is +1, and the upstream or downstream locations of the variation are shown relative to the 5’ nucleotide. (C) Illustration of substitution types for SNPs that occurred in sheep pri-miRNAs. Arrows indicate the direction of the single nucleotide polymorphism.

Several trends can be observed in Tables 1 and 2. Firstly, most of the 22 pri-miRNAs have less than three genetic variations (Fig. 2A). The pri-miR-101–2 has the most SNPs (seven SNPs), followed by pri-let-7a (five SNPs) and pri-miR-27b (four SNPs). Only six pri-miRNAs have deletions/insertions. These results indicated that some miRNA genes could have been under strong selection pressure during sheep evolution. If the levels of variation in pri-miRNA sequences were too high for their role in processing and maturation to be maintained, the expression of their target genes would also be affected. This, in turn, could influence the natural physiological activities of sheep and perhaps prevent adaptation to the environment, which may lead to the extinction of different breeds.

Secondly, further analysis of the location of genetic variations showed that most were distributed in the two flanking regions of pre-miRNA, ranging from 100 nt to 300 nt up- or downstream of the pre-miRNA. Only two SNPs (g.94625852G>A in pre-miR-29a and g.27314935G>A in pre-Let-7a) were detected in pre-miRNA and no genetic variations were detected in mature miRNA. More genetic variation, particularly deletions/insertions, appeared downstream of pre-miRNA, compared with upstream. Six deletions/insertions were detected in downstream regions (Fig. 2B). We speculated that the genetic variations in pre-miRNA and mature miRNA have significant influence on pre-miRNA processing and target identification and many may have been eliminated during sheep evolution.

Finally, the base transition and transversion rates were analyzed. We found that the most frequent substitutions are T>C (13/41), C>T (7/41) and G>A (7/41) (Fig. 2C), which are similar to the results obtained in rice [22, 23].

### Effect of genetic variations on pri-miRNA stability and secondary structure

**Fig 3:**
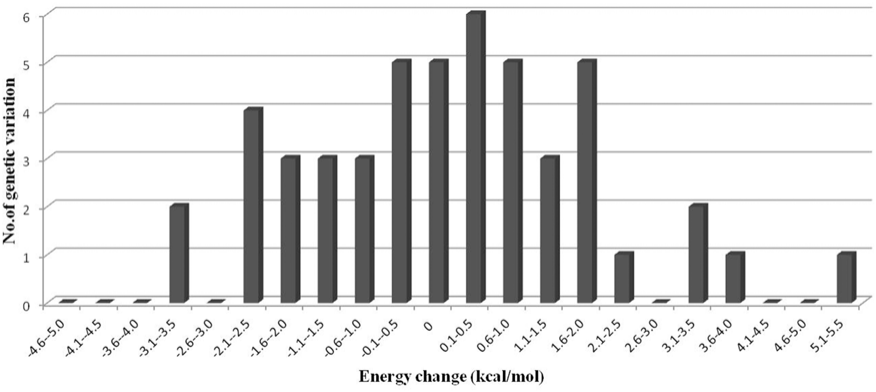
The energy change distribution of secondary structures associated with genetic variations.

Both pri-miRNA and pre-miRNA usually contain several hair-pin secondary structures that are important for identification and processing by Drosha and Dicer [24, 25]. In order to investigate the effect of genetic variations on pri-miRNA stability and secondary structures, the RNAstructure program (Version 5.6, July 2013, Mathews Lab) was used to predict optimal secondary structures of wild type pri-miRNAs and those with genetic variations. The energy changes of the hairpin structures of the pri-miRNAs caused by the genetic variations were also calculated to compare their stabilities (Fig. 3 and Table 3).

**Table 3:** The effects of genetic variations on the energy of pri-miRNA secondary structures.

		Energy △G (kcal/mol)		
MiRNA	Genetic variation	Wild type	Genetic variation type	Energy change
				△△G (kcal/mol)
miR-133a	A>G	-236.0	-239.2	-3.2
	CCC	-236.0	-237.6	-1.6
miR-133b	C>T	-189.7	-190.1	-0.4
miR-206	T>C	-242.4	-240.4	2.0
	C>A	-242.4	-243.8	-1.4
	C>T	-242.4	-242.0	0.4
miR-99a	C>T	-146.7	-144.9	1.8
	T>G	-146.7	-146.8	-0.1
	G>A	-146.7	-146.3	0.4
	ATATATATATATAC	-146.7	-144.9	1.8
	ATATATATATATATAC	-146.7	-143.6	3.1
miR-29a	T>G	-183.7	-183.4	0.3
	G>A	-183.7	-182.2	1.5
	G>A	-183.7	-183.6	0.1
	CTT	-183.7	-182.8	0.9
	TAATAATAC	-183.7	-182.2	1.5
miR-199a	T>C	-223.7	-222.9	0.8
	T>C	-223.7	-225.3	-1.6
miR-378–2	G>T	-180.9	-180.9	0
miR-27b	T>C	-262.9	-262.6	0.3
	G>C	-262.9	-259.4	3.5
	G>C	-262.9	-259.0	3.9
	T>A	-262.9	-260.9	2.0
miR-29c	A>G	-203.0	-204.4	-1.4
	G>T	-203.0	-202.6	0.4
	T>C	-204.6	-203.0	1.6
	CA	-203.0	-203.3	-0.3
miR-101–2	T>C	-174.8	-177.0	-2.2
	C>T	-174.8	-176.9	-2.1
	T>A	-174.8	-174.2	0.6
	T>C	-174.8	-173.9	0.9
	G>A	-174.8	-174.8	0
	G>A	-174.8	-174.8	0
	A>G	-174.8	-175.5	-0.7
miR-140	T>G	-256.0	-256.7	-0.7
	C>T	-256.0	-256.9	-0.9
miR-143	G>C	-238.2	-238.3	-0.1
miR-128–1	G>A	-120.6	-119.8	0.8
	ATCATGC	-120.6	-121.1	-0.5
miR-128–2	T>C	-235.3	-233.7	1.6
	T>C	-235.3	-234.2	1.1
	T>C	-235.3	-230.2	5.1
Let-7a	T>C	-159.4	-157.3	2.1
	G>A	-159.4	-159.4	0
	T>C	-159.4	-161.5	-2.1
	T>C	-159.4	-160.5	-1.1
	C>T	-159.4	-162.9	-3.5
	T	-159.4	-159.4	0
Let-7c	T>C	-177.7	-179.9	-2.2
Average absolute energy change (|△△G|)				1.4

**Fig 4:**
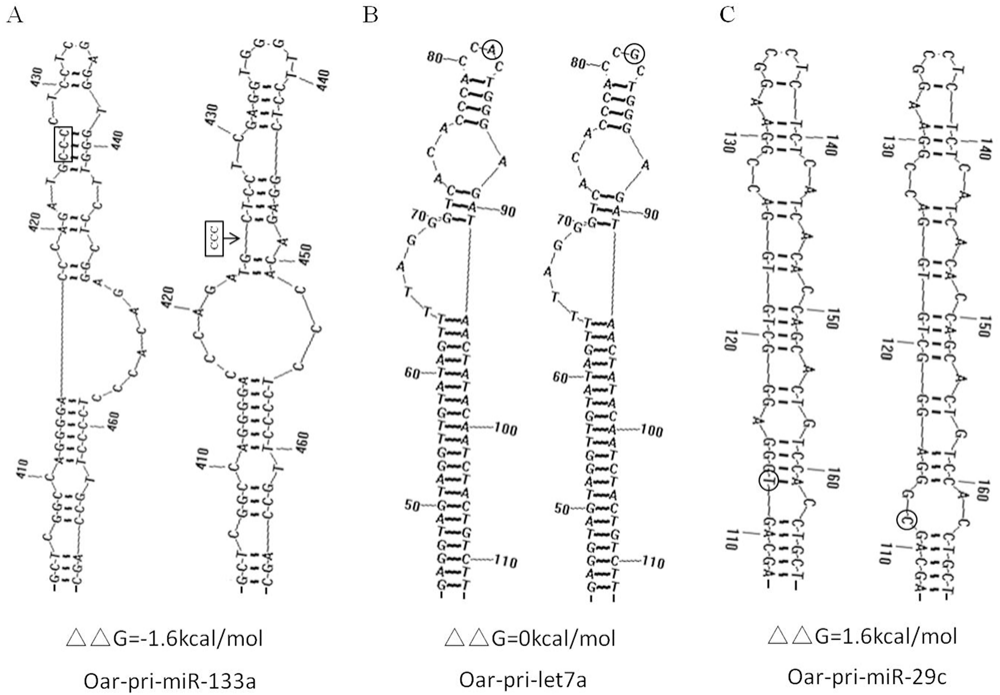
Illustration of pri-miR-133a (A), pri-let7a (B) and pri-miR-29c (C) secondary structure changes caused by genetic variations. The box in (A) and circles in (B) and (C) mark the position of the genetic variations. Three typical pri-miRNAs secondary structures (parts of them) were found to display increased (ΔΔG = −1.6 kcal/mol), unchanged (ΔΔG = 0 kcal/mol) and decreased stability of hairpin structures (ΔΔG = 1.6 kcal/mol) as a result of the genetic variations.

The results of secondary structure and energy change analysis showed that only 10.2% (5/49) of genetic variations have no effect on these parameters. These variations are generally located in the loop of the hair-pin structure and do not generate new base pairing (Fig. 3 and 4B). In contrast, 89.8% (44/49) of variations altered both the secondary structures and energy, with further analysis indicating that 54.5% (24/44) of these variations caused an increase in energy of pri-miRNA secondary structures. This has the effect of making the structure more unstable, which may affect the mature processing of pri-miRNA and alter the level of mature miRNA (Figs. 3 and 4C). A reduction in energy and increase in hair-pin stability was observed in 45.5% (20/44) of pri-miRNAs with genetic variations that generated new base pairing (Figs. 3 and 4A).

The maximum and minimum absolute energy changes were 5.1 kcal/mol (T>C in pri-miR-128–2) and 0.1 kcal/mol (T>G in pri-miR-99a), respectively. The alteration of the hair-pin structures of these pri-miRNAs corresponded to the change in energy; the more the absolute energy changed, the more the structure was affected [10]. On average, the absolute energy change of pri-miRNA secondary structures (|ΔΔG|), caused by SNPs in sheep, was 1.4 kcal/mol, which is lower than that found in the human (2.1 kcal/mol) and rice genome (2.4 kcal/mol) [9, 10]. However, it is important to note that the number of genetic variations and pri-miRNAs analyzed in our article are fewer than those in the aforementioned experiments in the human and rice genomes; thus, the absolute energy changes of the pri-miRNA secondary structures (|ΔΔG|) may not be representative of the sheep genome.

### Allele and genotype frequencies of pri-miR-133a, pri-miR-29a and pri-miR-27b

**Fig 5:**
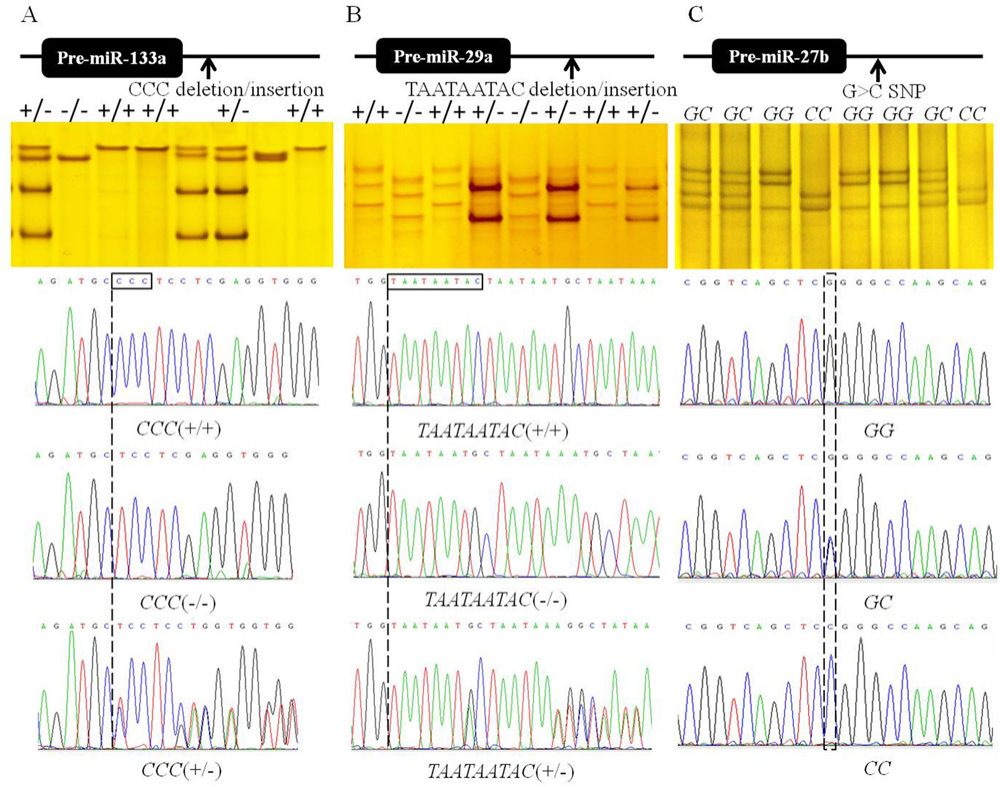
PCR-SSCP and sequencing of pri-miR-133a (A), pri-miR-29a (B) and pri-miR-27b (C). The boxes in the sequencing trace show the genetic variations. *CCC* (+/−) in (A) and *TAATAATAC* (+/−) in (B) appear mixed, with disordered peaks due to the deletion locus. *GC* in (C) appears as double peaks because the genetic variations occurred in one chromosome, but not the other.

When the allele and genotype frequencies of the 49 genetic variations in 16 pri-miRNAs mentioned above were further analyzed, some genetic variations of miRNA genes, just like the CCC deletions/insertions in pri-miR-133a, G>C SNP in pri-miR-27b, etc., differed significantly among sheep breeds. However, these results were obtained from only 160 samples from four sheep breeds (40 samples of each breed); thus, they may not be representative of the population. Recent studies have confirmed that miR-133a, miR-29a and miR-27b play important roles in skeletal muscle development [16, 17, 32, 33]. Thus, the allele and genotype frequencies of the CCC and TAATAATAC deletions/insertions in pri-miR-133a and pri-miR-29a, respectively, and the G>C SNP in pri-miR-27b were further investigated in large samples of five sheep breeds (Texel, Suffolk, Chinese Merino, Alaty and Hu sheep) to determine the association between the genetic variations and meat production performance (Fig. 5 and Table 4).

**Table 4:** Allele and genotype frequencies of pri-miR-133a, pri-miR-29a and pri-miR-27b in five sheep breeds.

		Breeds				
Genomic variation		Suffolk sheep	Texel sheep	Altay sheep	Chinese Merino	Hu sheep
CCC deletion/insertion in pri-miR-133a	Number	120	100	150	115	155
	Genotype frequency					
	*CCC*(+/+)	1(120)	0.32(32)	0.07(10)	0.04(5)	0.03(4)
	*CCC*(+/-)	0(0)	0.60(60)	0.16(24)	0.17(20)	0.16(25)
	*CCC*(-/-)	0(0)	0.08(8)	0.77(116)	0.78(90)	0.81(126)
	Allele frequency					
	*CCC*(+)	1	0.62	0.15	0.13	0.13
	*CCC*(-)	0	0.38	0.85	0.87	0.87
	Hardy-Weinberg test					
	χ^2^		7.47*	19.53**	6.26*	0.68
TAATAATAC deletion/insertion in pri-miR-29a	Number	117	95	145	113	140
	Genotype frequency					
	*TAATAATAC* (+/+)	0.03(3)	0.36(34)	0.14(20)	0.03(3)	0.04(6)
	*TAATAATAC* (+/-)	0.59(69)	0.50(48)	0.56(81)	0.52(59)	0.73(102)
	*TAATAATAC* (-/-)	0.38(45)	0.14(13)	0.30(44)	0.45(51)	0.23(32)
	Allele frequency					
	*TAATAATAC* (+)	0.30	0.61	0.42	0.29	0.41
	*TAATAATAC* (-)	0.70	0.39	0.58	0.71	0.59
	Hardy-Weinberg test					
	χ^2^	20.51**	0.37	3.21	8.49*	36.30**
G>C SNP in pri-miR-27b	Number	120	98	149	115	150
	Genotype frequency					
	*GG*	0.33(40)	0.12(12)	0.47(70)	0.01(2)	0.31(47)
	*GC*	0.21(25)	0.51(50)	0.15(22)	0.09(10)	0.49(73)
	*CC*	0.46(55)	0.37(36)	0.38(57)	0.90(103)	0.20(30)
	Allele frequency					
	*G*	0.44	0.38	0.54	0.06	0.56
	*C*	0.56	0.62	0.46	0.94	0.44
	Hardy-Weinberg test					
	χ^2^	39.91**	0.72	73.52**	5.41	0.03

Note: The numbers in the brackets are the number of individuals with the genotype

*P < 0.05 (χ^2^_0.05_ = 5.99)

**P < 0.01 (χ^2^_0.01_ = 9.21).

As shown in Fig. 5, the *CCC* (+/+), *CCC* (+/−) and *CCC*(−/−) genotypes in pri-miR-133a, *TAATAATAC* (+/+), *TAATAATAC* (+/−) and *TAATAATAC* (−/−) genotypes in pri-miR-29a and *GG*, *CC* and *GC* genotypes in pri-miR-27b were detected in five sheep breeds. The sequencing traces for *CCC* (+/−) and *TAATAATAC* (+/−) appear mixed and the peaks were disordered (Fig. 5A and B) because nucleotides were deleted from one copy of the gene but not the other.

Table 4 indicates that only *CCC* (+/+) in pri-miR-133a was detected in 120 Suffolk sheep. The *CCC* (+/-) and *CCC* (−/−) genotypes were not detected, which is significantly different from in the Altay sheep, Chinese Merino and Hu sheep, in which *CCC* (−/−) is the dominant genotype. The Suffolk and Texel sheep are famous for excellent meat production compared with the other three sheep breeds. The *CCC* (+/−) and *CCC* (−/−) genotypes were also detected in Texel but the meat production traits of this breed have already been shown to be due to another genetic mechanism [20]. The Suffolk and Hu sheep are in a state of Hardy–Weinberg equilibrium (*P* > 0.05) at this locus, whilst the Texel and Chinese Merino sheep populations deviated from Hardy–Weinberg equilibrium (*P* < 0.05), and the Altay sheep deviated significantly (*P* < 0.01).

The allele and genotype frequencies of the TAATAATAC deletion/insertion (g.94625593-g.94625601) in pri-miR-29a and the G>C SNP (g.31138951) in pri-miR-27b also showed differences between the five sheep breeds. However, these loci were not associated with meat production performance. We speculated that there may be two reasons for this result; 1) these genetic variations do not affect miRNA biogenesis or 2) the miRNA biogenesis is affected but the targets of the miRNA are not essential for the regulation of muscle development and growth. The results of the cell assay and qRT-PCR showed that the levels of mature miR-29a were significantly altered by the TAATAATAC deletion/insertion (g.94625593-g.94625601) but, because the targets of miR-29a are unclear in sheep skeletal muscle, we cannot explain the functions of this genetic variation.

### Alteration of mature miRNA

**Fig 6:**
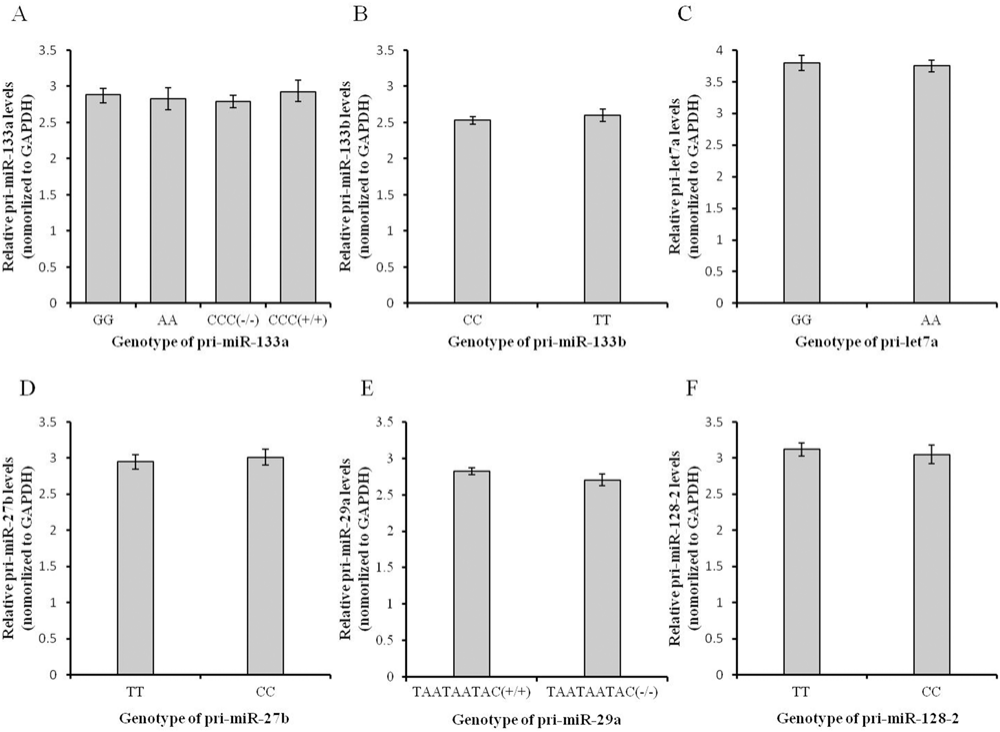
Relative expression levels of pri-miR-133a (A), pri-miR-133b (B), pri-let7a (C), pri-miR-27b (D), pri-miR-29a (E) and pri-miR-128–2 (F) among the different genotypes of pri-miRNAs. GAPDH (glyceraldehyde-3-phosphate dehydrogenase) was chosen as the internal control gene. Seven genetic variations of six pri-miRNAs with different energy changes were chosen to investigate the effect of these energy changes on pri-miRNA expression in transfected HeLa cells. The expression level of each pri-miRNA showed no significant difference between allelic variants. Graphs were generated using MS Excel. Bar graphs show the mean ± SEM.

**Fig 7:**
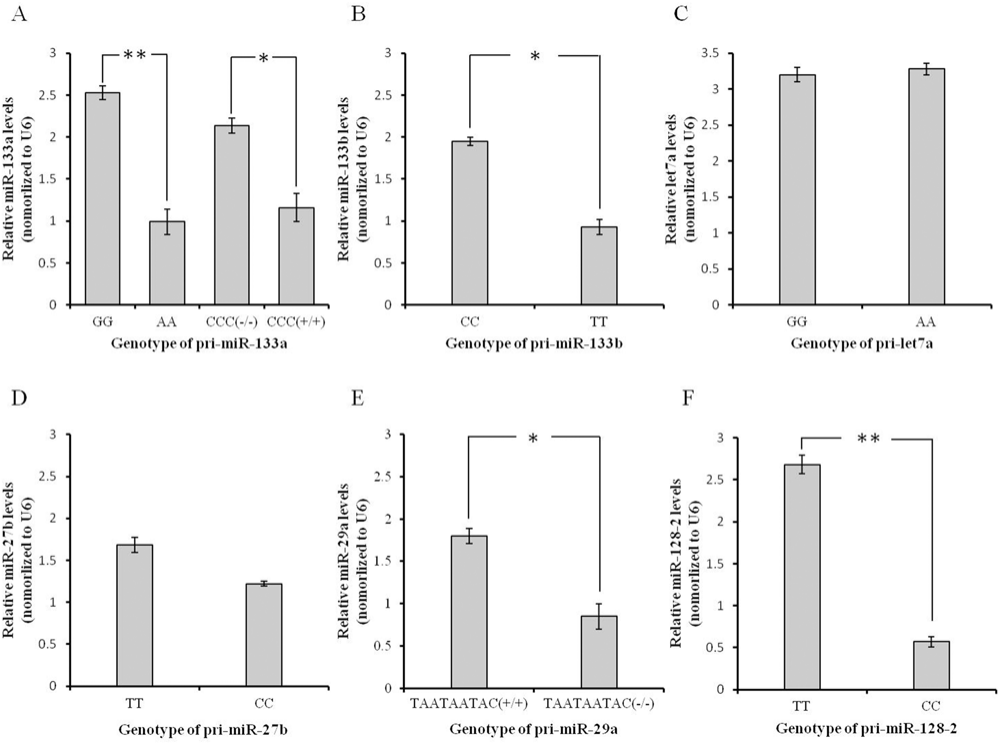
Relative expression levels of mature miR-133a (A), miR-133b (B), let7a (C), miR-27b (D), miR-29a (E) and miR-128–2 (F) among the different genotypes of pri-miRNAs. miRNA biogenesis was affected in relation to the degree of energy change caused by the genetic variation. Graphs were generated using MS Excel. Bar graphs show the mean ± SEM.

The effects of genetic variations on miRNA biogenesis were investigated with a cell assay and qRT-PCR. The results are shown in Figs. 6 and 7. We confirmed that the expression level of each pri-miRNA is not significantly different between allelic variants (Fig. 6). If the level of mature miRNA is changed, we can assume that the genetic variations affect the miRNA biogenesis processing.

The results of the mature miRNA qRT-PCR showed that the expressions of miR-133a and miR-128–2 were significantly altered (*P* < 0.01) by the A>G (g.54047491) and T>C (g.9630372) SNPs, respectively (Fig. 7A, F). The levels of miR-133a, miR-133b and miR-29a were also changed (*P* < 0.05) by the CCC deletion/insertion (g.540047592-g.540047594), C>T SNP (g.24358041) and TAATAATAC deletion/insertion (g.94625593-g.94625601), respectively (Fig. 7A, B, E). The G>A SNP (g.27314935) in pri-let7a and T>C SNP (g.31139368) in pri-miR-27b did not significantly affect their biogenesis processing (*P* > 0.05) (Fig. 7C, D). These results have led us to speculate that if the energy of the hair-pin structure is increased, then the levels of mature miRNAs are decreased, and vice versa. Furthermore, the greater the energy change, the more the level of mature miRNA is affected.

In addition, the |△△G| of pri-miR-133b and pri-miR-27b were 0.4 kcal/mol and 0.3 kcal/mol, respectively. The difference is only 0.1 kcal/mol but their influence on miRNA biogenesis is different. The C>T SNP of pri-miR-133b significantly affected the biogenesis of miR-133b (*P* < 0.05) but the T>C SNP of pri-miR-27b did not influence the biogenesis (Fig. 7B, D). The location of the C>T SNP in pri-miR-133b is +80, which is closer to the mature miRNA than the T>C SNP in pri-miR-27b (−261). We believe that the location of the genetic variation has an important influence on miRNA biogenesis, with the effect increasing with proximity to the mature miRNA. More data is required to confirm this.

## Discussion

### miRNA transcriptome profiles in sheep skeletal muscle

There is increasing evidence to indicate that miRNAs play important roles in the regulation of gene expression [7, 26, 27, 28]. Identification of miRNA transcriptome profiles in different tissues of organisms is the first step to investigate their functions. In livestock husbandry, improvement of meat production is an important goal, and clarification of miRNA expression and function in skeletal muscle will be an effective approach to achieve this aim. Skeletal muscle miRNA transcriptome profiles of common livestock, such as bovine, swine and horse, are already available [19, 20, 21, 29]. We are the first to analyze miRNA expression in sheep skeletal muscle and we have identified 261 miRNAs using microarray methodology. We compared our results to the data of bovines, swine and horses, and found that the types and expression levels of most miRNAs expressed in the skeletal muscle of these animals, especially the highly expressed miRNAs, were similar [20, 21, 29]. Thus, we speculated that these miRNAs may play important roles in skeletal muscle development, and have been under strong selective pressure during evolution.

We further analyzed the microarray result and found that only 22 miRNAs; let-7a, let-7c, miR-1, miR-206, miR-133a, miR-133b, miR-128–1, miR-128–2, miR-140, miR-143, miR-199a, miR-22, miR-26a, miR-27b, miR-29a, miR-29c, miR-378–1, miR-378–2, miR-486, miR-499, miR-99a and miR-101–2 have high levels of expression. We hypothesized that these miRNAs are important to sheep skeletal muscle development. In fact, the functions of these miRNAs in skeletal muscle differentiation and proliferation have already been researched extensively in recent years. The miRNAs miR-1 and miR-133 are specifically expressed in adult cardiac and skeletal muscle tissues, but not in other tested tissues [30]. They have distinct roles in skeletal muscle proliferation and differentiation: miR-1 promotes myoblast differentiation, whereas miR-133 stimulates myoblast proliferation [16]. The miRNA miR-133 is a key regulator of vascular smooth muscle cells and can control the vascular smooth muscle cell phenotypic switch, in vitro, and vascular remodeling, in vivo. It therefore has the potential to be a therapeutic target in vascular diseases [31]. The miRNA miR-29 can reduce proliferation and facilitate differentiation of myoblasts in skeletal muscle development by targeting Akt3, which is a member of the serine/threonine protein kinase family that is responsive to growth factor cell signaling [32]. The miRNA mir-27b can regulate the expression of myostatin and may have a relationship in Piedmontese cattle, which have the double-muscled phenotype [33]. In Texel sheep, miR-1 and miR-206 also target myostatin and cause the double-muscled phenotype in this breed of sheep [34].

The targets and functions of many miRNAs mentioned above remain unclear in sheep and more studies are required.

### The genetic variations of pri-miRNAs

MicroRNAs are key regulators of gene expression and simultaneously affect a multitude of genes. Hence, a small genetic change in a miRNA sequence can theoretically lead to widespread phenotypic effects [10]. Abelson *et al*. first demonstrated that miRNA-related SNPs can affect phenotype when they found that a mutation in the miR-189 binding site of the *SLITRK1* gene is associated with Tourette’s syndrome [35]. Since this finding, the genetic variations of miRNA genes have been widely researched and many genetic changes have been shown to have important functions [8, 10, 11, 12, 13].

Here, we investigated the genetic variations of 22 pri-miRNAs expressed specifically or at a high level in sheep skeletal muscle. In 16 pri-miRNAs, a total of 49 genetic variations, which included 41 SNPs and eight deletions/insertions, were identified in five breeds of sheep for the first time. There were six pri-miRNAs, such as pri-miR-499, pri-miR-26a and pri-378–1, in which no genetic variations were observed in sheep. We speculated that these miRNA genes are under strong negative selection pressures and the same phenomenon has been found in studies of humans, rice and *Arabidopsis* [9, 10, 36].

Amongst the 49 genetic variations, none were located in mature miRNA sequence, two were found in pre-miRNAs and the rest were detected in the flanking regions. This implied that the mature miRNAs and pri-miRNA sequences are more conserved. Genetic variations in mature miRNAs may render the miRNA unable to bind to the target or may generate a new target, which changes the normal biological activities and causes disease. In fact, many genetic variations associated with cancer are located in the seed sequence of mature miRNA [11, 12, 13]. The variations in pre-miRNAs and flanking regions may only alter the levels of miRNAs and do not influence the target recognition, which allows genetic variations to become concentrated in these regions [8, 36, 37].

In contrast, a large number of SNPs have been detected in seed sequences in humans and rice [9, 10]. The major reason underlying the observed inconsistencies may be that we investigated only 22 miRNAs. With such a small sample size, some genetic variations in mature miRNA may not have been detected. Another reason may be that the sheep involved in our research were healthy and most genetic variations found in seed sequences are associated with disease. More miRNAs should been investigated in future studies.

### The effect of genetic variations on miRNA biogenesis and function

Genetic variations in miRNA genes are thought to affect function in one of three ways: first, through the transcription of the primary transcript; second, through pri-miRNA and pre-miRNA processing; and third, through their effects on miRNA-mRNA interactions [8]. As the 49 genetic variations discussed here were all located in pre-miRNA and flanking regions, we speculated that they function mainly by the first two methods.

The genetic variations may change the stability and hair-pin structure of pri-miRNA, which is important to Drosha binding and cutting, and thus affects the processing of the pri-miRNA [24, 25]. Sun G *et al*. have confirmed that the minimum energy change that can affect the production of mature miRNAs is 0.3 kcal/mol [38], which is similar to our results obtained in a cell assay and qRT-PCR. Our data indicated that there is a relationship between the genetic variation and miRNA biogenesis processing whereby the more the genetic variation changes the energy of the secondary structure, the more the expression level is affected. If the genetic variation decreases the stability of the pri-miRNA hair-pin structure (an energy rise) then the expression level will be reduced, and vice versa. There may be some exceptions, so more experimental data are needed to validate this result.

The miRNAs investigated here are expressed specifically or at a high level in sheep skeletal muscle but not all of the genetic variations are associated with the meat production performance of sheep. In larger samples of five sheep breeds, three genetic variations were further studied but only the *CCC* deletion/insertion of pri-miR-133a was determined to be significantly associated with sheep meat production. A subsequent cell assay also confirmed that it influences the levels of mature miR-133a (Table 4 and Fig. 7A). Research in pigs has also revealed that SNPs in miR-1, miR-133b and miR-206 affect the muscle fiber and meat quality traits [39, 40]. These three miRNAs are muscle specific and we speculated that the targets of miR-1, miR-133b and miR-206 are crucial to myoblast differentiation and proliferation. Genetic variations in these three miRNAs have significant influence on sheep meat production traits. Although some genetic variations, such as the TAATAATAC deletion/insertion (g.94625593-g.94625601) in pri-miR-29a (Table 4 and Fig. 7E), changed the mature miRNA level, they do not affect the meat traits. It is possible that their targets are not important for muscle growth and further research is required into miRNA targets.

In conclusion, we have produced the first miRNA transcriptome profile from sheep skeletal muscle and have identified 22 miRNAs that are expressed specifically or at a high level. We applied PCR-SSCP and sequencing to identify 49 genetic variations, which included 41 SNPs and eight deletions/insertions in 16 of the 22 miRNAs. The characteristics and functions of the miRNAs were further investigated by a cell assay and qRT-PCR. As discussed above, there are over 300 miRNAs expressed in sheep skeletal muscle and although they are expressed at a lower level than the 22 discussed in detail, some may also play an important role in sheep muscle growth and development. Their genetic variations and functions should also be studied in the future to obtain a more comprehensive understanding of genetic variations and their functions in sheep. Here, we focused on the effect of genetic variations on miRNA biogenesis but the targets of these miRNAs will be confirmed in our subsequent studies. Our present findings are a good starting point and will be helpful to related research.

## Materials and Methods

### Ethics statement

All animal experimental procedures were approved by the Biological Studies Animal Care and Use Committee, Xinjiang Production and Construction Corps, Peoples Republic of China, and the ethics committee of the Xinjiang Academy of Agricultural and Reclamation Sciences. The ethics committee of the Xinjiang Academy of Agricultural and Reclamation Sciences, P. R. China approved this study (Permit Number: XAARS-AE-2012015).

### miRNA microarray and data analysis

Biceps femoris muscle samples were collected from three healthy adult (12-month-old) Altay sheep. The sheep were anaesthetized by intravenously injecting Pelltobarbitalum Natricum (30 mg/kg body weight, Ningbo, China) and then slaughtered. The biceps femoris muscle samples were collected immediately, and snap-frozen in liquid nitrogen, then stored in -80°C. Total RNA was extracted using TRIzol (Invitrogen, Carlsbad, CA, USA). After quantification (determined using an Agilent 2100 bioanalyzer), the RNA was labeled using the miRCURY Array Power Labeling kit (Cat #208032-A, Exiqon, Denmark) in accordance with the manufacturer's protocol.

The microarray used was the Exiqon microRNA Array V.9.2 (Exiqon, Denmark), and hybridizations were performed in accordance with the manufacturer’s protocol. In brief, 12.5 μl of labeled RNA was mixed with 90 μl of 1.5× hybridization buffer and 77.5 μl of nuclease-free H_2_O. The reaction was incubated at 95°C for 2 min and protected from light. The solution was left on ice for at least 2 min and then spun briefly. The hybridization assembly was inserted into a 3.1 × 9 cm heat-shrunk hybridization bag and hybridized over night at 56°C. The slide was washed using the miRCURY Array Wash buffer kit (Cat #208021, Exiqon) according to the manufacturer's instructions: wash with buffer A for 5 min, B for 2 min, C for 2 min, followed by a final brief wash in water. The slide was then dried by centrifugation for 5 min at 200 × *g* (1,000 rpm). The slides were scanned immediately after drying using the GenePix 4000B Scanner (Molecular Devices, Sunnyvale, CA, USA). Data extraction was performed using GenePix Pro6.0 and data were exported to Excel. For each spot, the signal was verified by subtracting the background intensity (B) from foreground intensity (F). The verified signal value = F – B. The control spots were filtered. If the verified signal value of one spot was more than 50, then this signal was considered to be valid. The array dataset was submitted to ArrayExpress and the accession number was obtained (E-MTAB-3096).

The accuracy of the microarray data was validated using qRT-PCR. Five highly expressed miRNAs (miR-1, miR-206, miR-133, miR-29 and let-7) and three lowly expressed miRNAs (miR-155, miR-15 and miR-146) were chosen, and their expression levels in the biceps femoris muscle of adult (12-month-old) Altay sheep were determined using qRT-PCR according to an approach called miR-Q [41]. Briefly, total RNA extracted from the biceps femoris muscle was reverse transcribed by M-MLV reverse transcriptase (Invitrogen, Carlsbad, CA, USA) and specific primers with a stem-loop structure (S3 Table), in accordance with the manufacturer's protocol. Quantitative PCR was performed with two primers (S3 Table) to detect the levels of the miRNAs. The levels of mature miRNAs were normalized to those of U6, which was reverse transcribed and amplified by PCR with the U6 primers (S3 Table).

The fold difference in expression of miRNAs was calculated using the equation 2^△△ct^, where △Ct = Ct (miRNA) − Ct (U6) and △△Ct = △Ct miRNA1 − △Ct miRNA2. A *P*-value <0.05 was considered to be statistically significant. The graphs were generated with MS Excel. Bar graphs show the mean ± SEM.

### Animals and genomic DNA isolation

We chose five sheep breeds with different meat production performances to investigate the relationship of the genetic variations in the pri-miRNAs and the meat production traits. Suffolk and Texel sheep are famous sheep breeds with perfect meat production performance; Altay and Hu sheep are Chinese famous local breeds with excellent fat deposition ability; and Chinese Merino is a Chinese fine wool sheep breed, although the meat production traits of Altay, Hu and Chinese Merino sheep are poorer than those of Suffolk and Texel sheep.

Blood samples (10 ml taken from the jugular vein and mixed with acid citrate dextrose (ACD) anticoagulant) were collected from 120 Suffolk sheep (Xin-ao Sheep Breeding Farm, Manasi County, Xinjiang Uygur Autonomous Region, PR China), 150 Altay sheep (Fuyun County, Xinjiang Uygur Autonomous Region, PR China), 115 Chinese Merino sheep (Ziniquan Sheep Breeding Farm, Shihezi City, PR China), 100 Texel sheep (Xin-ao Sheep Breeding Farm, Manasi County, Xinjiang Uygur Autonomous Region, PR China) and 155 Hu sheep (Hailun sheep incorporated company, Taizhou City, Jiangsu Province, PR China).

Genomic DNA was extracted from 0.5 ml of whole blood using phenol–chloroform. It was stored in TE buffer (10 mmol/l Tris–HCl [pH 8.0], 1 mmol/l EDTA [pH 8.0]) at-20°C.

### Primers and amplification of pri-miRNAs

The 22 sheep pri-miRNA sequences were obtained from the miRBase database (http://www.mirbase.org/) and the UCSC Genome Browser (http://genome.ucsc.edu/). Three pairs of primers were designed to amplify the pre-miRNAs, and the upstream and downstream sequences of the flanking regions. The amplicon produced from each primer pair was approximately 300 base pairs (bp) to provide a suitable template for SSCP. The sequences of all primers are given in S4 Table.

The PCR reactions were performed by mixing 2.5 μl of reaction buffer, 2 μl of dNTPs (2.5 mM each), 0.5 μl (10 μM) each of the forward and reverse primers, 0.5 μl (5 U/μl) of Taq DNA polymerase (TaKaRa, Dalian, China), 1 μl of sheep genomic DNA and 18 μl of hyper-pure water, in a final volume of 25 μl. The cycling was performed on a Mastercycler-5333 (Eppendorf AG, Hamburg, Germany), in accordance with the following program: 94°C for 5 min, 30 cycles of 94°C for 30 s, annealing at an optimum temperature for 30 s (the optimum annealing temperature of each primer are shown in S4 Table), and 72°C for 30 s. A final extension of 72°C for 10 min was used. The PCR products were detected using 1.5% agarose gel electrophoresis.

### Genotyping and sequencing

One hundred and sixty sheep genomic DNA samples, which represented four different sheep breeds (40 Texel, Suffolk, Altay and Hu sheep), were amplified by PCR, and SSCP was employed to detect the polymorphisms present in each PCR product, in accordance with the following protocol: 2 μl of PCR product was mixed with 8 μl of denaturing sample loading buffer. The reaction was incubated at 98°C for 10 min and then placed on ice for 5 min. The 10 μl sample was then loaded onto a 10% polyacrylamide gel and electrophoresed at 300 V for 5 min, followed by 110 V for 16 h at 4°C–15°C. Silver staining was used to visualize and distinguish the different genotypes.

The PCR products that contained different genetic variations were chosen from the SSCP results, separated on a 1.5% agarose gel, purified with Agarose Gel PCR Fragment Clean-up system (Promega, Madison, WI, USA) and sequenced in both directions using Sanger sequencing (BGI, Beijing, China).

### Plasmid constructs and cell transfections

To investigate the effects of the genetic variations on pri-miRNA processing and maturation, a cell assay and qRT-PCR were employed to determine whether the level of mature miRNA was affected by these genetic variations. These methods were also used to analyze the relationship between the changes in the miRNA levels and the changes in energies of the pri-miRNA secondary structures caused by genetic variations. Six pri-miRNAs with different levels of energy changes, pri-miR-133a (g.54047491 A>G SNP, ΔΔG = −1.6 kcal/mol and g.540047592 CCC deletion/insertion, ΔΔG = −1.6 kcal/mol), pri-miR-133b (g.24358041 C>T SNP, ΔΔG = −0.4 kcal/mol), pri-let7a (g.27314935 G>A SNP, ΔΔG = 0 kcal/mol), pri-miR-27b (g.31139368 T>C SNP, ΔΔG = 0.3 kcal/mol), pri-miR-29c (g.94625593-g.94625601 TAATAATAC deletion/insertion, ΔΔG = 1.5 kcal/mol) and pri-miR-128–2 (g.9630372 T>C SNP, ΔΔG = 5.1 kcal/mol), were chosen for analysis by the assay described below.

The genomic regions that corresponded to the allelic variants of the pri-miRNAs were amplified from genomic DNA using gene-specific primers containing restriction enzyme cleavage sites. The primers sequences are given in S5 Table.

Amplification was conducted using the PCR system described above. The purified PCR fragments were cloned using the pGEM-T Easy Vector system (Promega), with reference to the manufacturer’s instructions. Clones were digested with restriction enzymes and ligated to the pcDNA3.1(+) vector (Invitrogen). All plasmid constructs were confirmed by sequencing.

HeLa cells (ATCC, Manassas, VA, USA) were cultured in Dulbecco’s modified Eagle medium (DMEM), which contained 5% fetal calf serum, 1% penicillin and streptomycin. When the HeLa cells were approximately 80% confluent, transient transfections were performed using electroporation technology (Lonza, Basel, Switzerland). A density of 1 × 10^6^ cells/transfection and 150 ng of plasmid were used, and the cells were cultured in a 5% CO_2_ incubator at 37°C.

### RNA analysis

HeLa cells were harvested after 48 h after transfection, and total RNA was extracted using TRIzol (Invitrogen, Carlsbad, CA, USA). To confirm whether the expression levels of the pri-miRNAs with different genetic variations were identical, or to show that the HeLa cells had not been transfected, a qRT- PCR method was applied, as described previously [37]. In brief, total RNA extracted from HeLa cells was reverse transcribed using M-MLV reverse transcriptase (Invitrogen) and specific primers (S6 Table) in accordance with the manufacturer's protocol. Quantitative PCR was conducted using a LightCycler2.0 instrument (Roche, Basel, Switzerland), with SYBR Green SuperMix (Qiagen, Valencia, CA, USA) and two specific primers (S6 Table), to detect the levels of the pri-miRNAs. The relative levels of the pri-miRNAs were calculated and normalized to the expression levels of the glyceraldehyde-3-phosphate dehydrogenase gene (GAPDH), which was reverse transcribed in accordance with the manufacturer’s protocol (Invitrogen) and amplified by PCR using GAPDH primers (S6 Table).

The levels of mature miRNAs were determined using an approach called miR-Q [41], as described above. The fold difference in expression of allelic forms was calculated using the equation 2^△△ct^, where △Ct = Ct (pri-miRNA or miRNA) − Ct (GAPDH or U6) and △△Ct = △Ct allele 1 − △Ct allele 2. A *P*-value <0.05 was considered to be statistically significant. Graphs were generated with MS Excel. Bar graphs show the mean ± SEM.

To avoid the genomic DNA from interfering with the results of the qRT-PCR, DNase I (TaKaRa, Dalian, China) was used to degrade the genomic DNA that contaminated the RNA samples.

### Statistical analysis

Allele and genotype frequencies of the CCC deletion/insertion (g.540047592-g.540047594) in pri-miR-133a, TAATAATAC deletion/insertion (g.94625593-g.94625601) in pri-miR-29a and G>C SNP (g.31138951) in pri-miR-27b were determined in five sheep breeds. The Hardy-Weinberg equilibrium was verified by calculating the expected frequencies and numbers, and was tested using the goodness-of-fit χ^2^ test. Significant differences in the levels of pri-miRNAs or mature miRNAs were determined using a one-way analysis of variance (ANOVA). *P*-values <0.05 were considered to be statistically significant. All statistical analyses were performed with Statistical Analysis System software (version 9.1.3; SAS Institute, Cary, NC, USA).

## Supporting Information

S1 Table Probe list for the microarray. (XLS)

S2 Table miRNAs detected in sheep skeletal muscle. (XLS)

S3 Table Primers used to reverse transcribe and quantify the levels of mature miRNAs by qRT-PCR. (DOCX)

S4 Table Primers used to amplify the genomic regions containing the allelic variants of pri-miRNAs for PCR-SSCP and sequencing. (DOCX)

S5 Table Primers used to amplify pri-miRNA regions for expression vector cloning. (DOC)
(DOCX)

S6 Table Primers used to reverse transcribe and quantify the levels of pri-miRNAs by qRT-PCR. (DOCX)
