# Supplementary material for: Identification and Analysis of Genetic Variations in Pri-MiRNAs Expressed Specifically or at a High Level in Sheep Skeletal Muscle
Source: PLoS One. 2015 Feb 20;10(2):e0117327. doi: 10.1371/journal.pone.0117327 (PMC4336289; doi:10.1371/journal.pone.0117327)
Supplement: S3 Table — (DOCX) [file pone.0117327.s003.docx]

**Table S3** **Primers used to reverse transcription and quantify the levels of mature miRNAs by qRT-PCR. (DOC)**

| MiRNA | Primers Name | Primers Sequence (5ˊ-3ˊ) | Note |
| --- | --- | --- | --- |
| miR-133a | miR-133aRTP | CTCGACTGAGTTGCCGTGAGTCGGCAACTCAGTCGAGACAGCTGG | Reverse Primer |
|  | miR-133aqU | TGAGTTGCCGTGAGTCGGCAACTC | aRT-PCR Primers |
|  | miR-133aqL | ACACTCCAGCTGGGTTGGTCCCCTTCAA |  |
| miR-133b | miR-133bRTP | CTCGACTGAGTTGCCGTGAGTCGGCAACTCAGTCGAGTAGCTGGT | Reverse Primer |
|  | miR-133bqU | TGAGTTGCCGTGAGTCGGCAACTC | aRT-PCR Primers |
|  | miR-133bqL | ACACTCCAGCTGGGTTTGGTCCCCTTCA |  |
| let7a | let7a-RTP | CTCGACTGAGTTGCCGTGAGTCGGCAACTCAGTCGAGAACTATAC | Reverse Primer |
|  | let7a-qU | TGAGTTGCCGTGAGTCGGCAACTC | aRT-PCR Primers |
|  | let7a-qL | ACACTCCAGCTGGGTGAGGTAGTAGGTT |  |
| miR-27b | miR-27bRTP | CTCGACTGAGTTGCCGTGAGTCGGCAACTCAGTCGAGGCAGAACT | Reverse Primer |
|  | miR-27bqU | TGAGTTGCCGTGAGTCGGCAACTC | aRT-PCR Primers |
|  | miR-27bqL | ACACTCCAGCTGGGTTCACAGTGGCTA |  |
| miR-29a | miR-29aRTP | CTCGACTGAGTTGCCGTGAGTCGGCAACTCAGTCGAGAACCGATT | Reverse Primer |
|  | miR-29aqU | TGAGTTGCCGTGAGTCGGCAACTC | aRT-PCR Primers |
|  | miR-29aqL | ACACTCCAGCTGGGTAGCACCATCTGA |  |
| miR-128-2 | miR-128-2RTP | CTCGACTGAGTTGCCGTGAGTCGGCAACTCAGTCGAGGAAAGAGA | Reverse Primer |
|  | miR-128-2qU | TGAGTTGCCGTGAGTCGGCAACTC | aRT-PCR Primers |
|  | miR-128-2qL | ACACTCCAGCTGGGTCACAGTGAACCGG |  |
| miR-155 | miR-155RTP | CTCGACTGAGTTGCCGTGAGTCGGCAACTCAGTCGAGACCCCTAT | Reverse Primer |
|  | miR-155qU | TGAGTTGCCGTGAGTCGGCAACTC | aRT-PCR Primers |
|  | miR-155qL | ACACTCCAGCTGGGTTAATGCTAATCGTG |  |
| miR-15 | miR-15RTP | CTCGACTGAGTTGCCGTGAGTCGGCAACTCAGTCGAGTGTAAACC | Reverse Primer |
|  | miR-15qU | TGAGTTGCCGTGAGTCGGCAACTC | aRT-PCR Primers |
|  | miR-15qL | ACACTCCAGCTGGGTAGCAGCACATCAAT |  |
| miR-146 | miR-146RTP | CTCGACTGAGTTGCCGTGAGTCGGCAACTCAGTCGAGACAGCCTA | Reverse Primer |
|  | miR-146qU | TGAGTTGCCGTGAGTCGGCAACTC | aRT-PCR Primers |
|  | miR-146qL | ACACTCCAGCTGGGTGAGAACTGAATTCCA |  |
| U6 | U6qU | CTCGCTTCGGCAGCACA | Reverse Primer  aRT-PCR Primers |
|  | U6qL | AACGCTTCACGAATTTGCGT |  |
