# Supplementary material for: Identification and Analysis of Genetic Variations in Pri-MiRNAs Expressed Specifically or at a High Level in Sheep Skeletal Muscle
Source: PLoS One. 2015 Feb 20;10(2):e0117327. doi: 10.1371/journal.pone.0117327 (PMC4336289; doi:10.1371/journal.pone.0117327)
Supplement: S4 Table — (DOCX) [file pone.0117327.s004.docx]

**Table S4 Primers used to amplify the genomic regions corresponding to allelic variants of pri-miRNAs for PCR-SSCP and sequencing.**

| Primers Name | Primers sequence (5ˊ-3ˊ) | Pri-miRNA | Product Size（bp） | Annealing Temperature (℃) |
| --- | --- | --- | --- | --- |
| pri-29c-162U-1 | AAGAAGAGCTTGAGAAGCAGAG | Pri-miR-29c | 289 | 58 |
| pri-29c-451L-1 | AGTCTGATTTCACCTACTCAGC |  |  | 58 |
| pri-29c-368U-2 | TCTCTCATCACACCAGCACTGT |  | 298 | 60 |
| pri-29c-666L-2 | TCTCTGCCCATTCATCTTCCAG |  |  | 60 |
| pri-29c-524U-3 | TTCTCCTGGTGTTCAGAGTCTG |  | 315 | 60 |
| pri-29c-839L-3 | AACAGCAACCAATTCGGAG |  |  | 55 |
| pri-27b-156U-1 | AGTAAACGAACCAAGCGGAAGG | Pri-miR-27b | 293 | 60 |
| pri-27b-449L-1 | TTCTTCTGCTTGCAGAGAGGAG |  |  | 60 |
| pri-27b-385U-2 | ATAGTTGCGCTCTCCGCAGTTC |  | 296 | 61 |
| pri-27b-681L-2 | ATCCTCCAGAATCCTGCTTGGC |  |  | 61 |
| pri-27b-579U-3 | TGCACCTGAAGAGAAGGTGAGA |  | 300 | 60 |
| pri-27b-879L-3 | TCACACATCATCTACCGCTGAC |  |  | 60 |
| pri-101-(2)136U-1 | AGCTATAGGAAAAAGGACTGCC | Pri-miR-101-2 | 297 | 58 |
| pri-101-(2)433L-1 | AGGTGGCTACAGAATGATCTTC |  |  | 58 |
| pri-101-(2)345U-2 | AGTAAGTGAGGTTGCTGTGAAC |  | 363 | 58 |
| pri-101-(2)708L-2 | CTTCCACCATCAACTTACAGGA |  |  | 58 |
| pri-101-(2)650U-3 | GAGACCAGAAGATTCTTCTGGA |  | 269 | 58 |
| pri-101-(2)919L-3 | GCTGTGCTAACTGTAAAGTCTC |  |  | 58 |
| pri-128-(1)97U-1 | TCTGTTGAAGCCTCTGTGTACC | Pri-miR-128-1 | 322 | 60 |
| pri-128-(1)419L-1 | AGCTAAACAGAGAACAGCCAGG |  |  | 60 |
| pri-128-(1)389U-2 | TTATGAGGCCTGGCTGTTCTCT |  | 229 | 60 |
| pri-128-(1)618L-2 | AAGGAAGCAGTGGAAACCTGAG |  |  | 60 |
| pri-128-(1)545U-3 | ATTTCTCACAGTGAACCGGTCT |  | 337 | 58 |
| pri-128-(1)882L-3 | ATGAATCTGGCATGCTTAGGAC |  |  | 58 |
| pri-128-(2)128U-1 | ACGGAGCGTTTGATTAACTCTG | Pri-miR-128-2 | 297 | 58 |
| pri-128-(2)425L-1 | TGTCAGTGACCTTCTTCTCAAG |  |  | 58 |
| pri-128-(2)349U-2 | AAGGCTAGGGAGTCATGTTAGG |  | 237 | 60 |
| pri-128-(2)586L-2 | GTGACACAGTAGGGAAAGAGAC |  |  | 60 |
| pri-128-(2)535U-3 | AGAGTGAGTAGCAGGTCTCACA |  | 299 | 60 |
| pri-128-(2)834L-3 | TCACGCATTACAGAGCCGCATA |  |  | 60 |
| pri-140-171U-1 | CAAAGCATTGTCTTGCCCTCACC | Pri-miR-140 | 343 | 62 |
| pri-140-514L-1 | AGGACACAGAGAGACAGACACAC |  |  | 62 |
| pri-140-427U-2 | TTTTCCGTGGTGACCGTGGTGA |  | 305 | 62 |
| pri-140-732L-2 | CCAGGTACAAGCTTCAAGCCAG |  |  | 62 |
| pri-140-674U-3 | CTGCTTGTGTAGCTCAAGCCTC |  | 265 | 62 |
| pri-140-939L-3 | CTTCCAGCCTACCTAAGCAGTC |  |  | 62 |
| pri-let-7a139U-1 | TTTCTCAAGGGAAGGATAAGTG | Pri-let7a | 296 | 56 |
| pri-let-7a435L-1 | TCTCCTAGGTAATCCTGGTTCT |  |  | 56 |
| pri-let-7a328U-2 | GGAAGGTTTGTTTCAGTTCCAC |  | 194 | 58 |
| pri-let-7a522L-2 | TACAACCTACTACCTCATCCCA |  |  | 58 |
| pri-let-7a455U-3 | AGCTGTGGTCCAATCTCAGCAT |  | 281 | 60 |
| pri-let-7a736L-3 | CCAATCCCCAATCAGTCTAGTC |  |  | 60 |
| pri-let-7c115U-1 | ACTTCTGTTCACATTCCAGTCG | Pri-let7c | 313 | 58 |
| pri-let-7c428L-1 | ACCTAAGGACTTAGCAAGGATG |  |  | 58 |
| pri-let-7c325U-2 | GGTTAGGATAGTCCTAGTACAC |  | 334 | 58 |
| pri-let-7c659L-2 | GACACATAACCTTCTTGCACAG |  |  | 58 |
| pri-let-7c592U-3 | AGCCATCGAGGAATTCTTCATC |  | 383 | 58 |
| pri-let-7c975L-3 | AAGACTCAGACAATGTGCTGTG |  |  | 58 |
| pri-22-103U-1 | TGATCCCTCCCATTTCTGGACC | Pri-miR-22 | 326 | 62 |
| pri-22-429L-1 | TATGTCACAGGGACACTGTCTC |  |  | 60 |
| pri-22-346U-2 | GAGCCAAGAAGCCCTTCATTAG |  | 306 | 60 |
| pri-22-652L-2 | TTCCAGAGAAGGGAGAAAGCAG |  |  | 60 |
| pri-22-556U-3 | CTGCCAGTTGAAGAACTGTTGC |  | 296 | 60 |
| pri-22-852L-3 | CTTCTTCCCACTGTCAAAGACC |  |  | 60 |
| pri-143-224U-1 | AAGCCCAACTCACCACTCTGTG | Pri-miR-143 | 328 | 62 |
| pri-143-552L-1 | TCCCAACTGACCAGAGATGCAG |  |  | 62 |
| pri-143-437U-2 | CTAATGCCCCTTCTCCTGCCTA |  | 267 | 62 |
| pri-143-704L-2 | GACTCAAGTCCAGTGCTTCCTC |  |  | 62 |
| pri-143-644U-3 | GGAAGGACGGGAGTGTTTCCAT |  | 202 | 62 |
| pri-143-846L-3 | GAAGGATTCTGAAGCCGCATC |  |  | 60 |
| pri-133aU-1 | TTGGTCACGTGACTGACCCTCAGAC | Pri-miR-133a | 193 | 65 |
| pri-133aL-1 | ACAGCAGCATCAACGATGAAGGCGG |  |  | 65 |
| pri-133aU-2 | TTGGTCCCCTTCAACCAGCTGTAGC |  | 246 | 65 |
| pri-133aL-2 | GTGATGCTGTGGTGTGCAGCAGACA |  |  | 65 |
| pri-206U-1 | ACCCCCACCCCAAAGCACCCTTATG | Pri-miR-206 | 187 | 67 |
| pri-206L-1 | TGCTTCCTCCATCGTCCGGATGCAG |  |  | 67 |
| pri-206U-2 | AGAGCACCTGGCCCCTTCTT |  | 164 | 62 |
| pri-206L-2 | GGGACCACAGTCAGTGTGCCTGATC |  |  | 62 |
| pri-99aU-1 | TATGCATCCTTAGAGCATTTTGGC | Pri-miR-99a | 213 | 56 |
| pri-99aL-1 | GTCAGATTACCACACTGACACAGA |  |  | 57 |
| pri-99aU-2 | TCTGTGTCAGTGTGGTAATCTGAC |  | 329 | 57 |
| pri-99aL-2 | CACGACTGGAATGTGAACAGAAGTT |  |  | 57 |
| pri-29aU-1 | CAACAGGTCAATGACGCAACATCTC | Pri-miR-29a | 304 | 60 |
| pri-29aL-1 | AACACGTTCTCTCTCCTTAGTCAG |  |  | 60 |
| pri-29aU-2 | AGAGAGAACGTGTTGGTGACCTTC |  | 207 | 62 |
| pri-29aL-2 | CCCTACAAAGTCTGTGGAATTCCTC |  |  | 62 |
| pri-199aU-1 | CCCAAACCTCCCCAGGGTGACATC | Pri-miR-199a | 221 | 65 |
| pri-199aL-1 | GTCTTGGGGAAAAGCCTCCCTTCCT |  |  | 64 |
| pri-199aU-2 | AGGAAGGGAGGCTTTTCCCCAAGAC |  | 279 | 65 |
| pri-199aL-2 | TGGCCCCTACTGTGCAAAACCTGTG |  |  | 65 |
| pri-378-2U-1 | ATGGAATGTAGCCCAGGGCCATTC | Pri-miR-378-2 | 188 | 64 |
| pri-378-2L-1 | CAGTAGATGACAGTGCTGCCCTGT |  |  | 64 |
| pri-378-2U-2 | ACAGGGCAGCACTGTCATCTACTG |  | 279 | 64 |
| pri-378-2L-2 | CTTGGCCTTTCTTCTCAGCTGCTG |  |  | 63 |
| pri-1-1U | GGTGGGATGAAATAGGGGAAAAGG | Pri-miR-1-1 | 378 | 60 |
| pri-1-1L | GCTGACACAGGCAAAGTGACAGAAC |  |  | 61 |
| pri-1-2U | CTGCAAATGATCTACCACTGCTCTC | Pri-miR-1-2 | 506 | 58 |
| pri-1-2L | GTGGAGACAGAAAATGGGTCACTTG |  |  | 59 |
| pri-486U | GGTGCCTGGGTGTGATGTGAAGTGC | Pri-miR-486 | 335 | 65 |
| pri-486L | GGAGACAGCAGCCCTGCGTGTTTGT |  |  | 66 |
| pri-499U | CCCCAAGTCCGGGGTGAAAGAGAAG | Pri-miR-499 | 459 | 63 |
| pri-499L | GGGGAGAAAGCACAAGAGGGCAGAG |  |  | 64 |
| pri-378-1U | GCCCTTCGGGTGATCTCTGTCGTC | Pri-miR-378-1 | 445 | 63 |
| pri-378-1L | GGGGGGAGGAGAGGAAGTTACAGG |  |  | 62 |
