# Supplementary material for: Identification and Analysis of Genetic Variations in Pri-MiRNAs Expressed Specifically or at a High Level in Sheep Skeletal Muscle
Source: PLoS One. 2015 Feb 20;10(2):e0117327. doi: 10.1371/journal.pone.0117327 (PMC4336289; doi:10.1371/journal.pone.0117327)
Supplement: S5 Table — (DOC) (DOCX) [file pone.0117327.s005.docx]

**Table S5** **Primers used to amplify pri-miRNAs regions for expression vector cloning.**

| Pri-miRNA | Primers Name | Primers Sequence (5ˊ-3ˊ) | restriction enzyme | Product Size（bp） |
| --- | --- | --- | --- | --- |
| Pri-miR-133a | miR-133aEU | CCCA**A**GCTTGGGTTTGGTCACGTGACTGACCCTC | HindⅢ | 551 |
|  | miR-133aEL | CCGGAATT**C**CGGTGGACACACACTCTCGGTGATG | EcoRⅠ |  |
| Pri-miR-133b | miR-133bEU | CCCA**A**GCTTGGGCATGTTAGTTCCTCACAATTGC | HindⅢ | 593 |
|  | miR-133bEL | CCGGAATT**C**CGGAAAGTCCTTACTGTGTGTCAAG | EcoRⅠ |  |
| Pri-let7a | let7aEU | CCCA**A**GCTTGGGGGAAGGTTTGTTTCAGTTCCAC | HindⅢ | 433 |
|  | let7aEL | CCGGAATT**C**CGGCCAATCCCCAATCAGTCTAGTC | EcoRⅠ |  |
| Pri-miR-27b | miR-27bEU | CCCA**A**GCTTGGGAGTAAACGAACCAAGCGGAAGG | HindⅢ | 747 |
|  | miR-27bEL | CCGGAATT**C**CGGTCACACATCATCTACCGCTGAC | EcoRⅠ |  |
| Pri-miR-29a | miR-29aEU | CCCA**A**GCTTGGGCGTTACACACAACAGGTCAATG | HindⅢ | 539 |
|  | miR-29aEL | CCGGAATT**C**CGGTTTTTCCTAGGTGTCCAGCATC | EcoRⅠ |  |
| Pri-miR-128-2 | miR-128-2EU | CCCA**A**GCTTGGGACGGAGCGTTTGATTAACTCTG | HindⅢ | 824 |
|  | miR-128-2EL | CCGGAATT**C**CGGTGGGGATTTGCACTCATTCTTG | EcoRⅠ |  |

Note: The sequence of restriction enzymes and protective bases were marked with underline, and the cut sites were indicated with red.
