# Supplementary material for: Identification and Analysis of Genetic Variations in Pri-MiRNAs Expressed Specifically or at a High Level in Sheep Skeletal Muscle
Source: PLoS One. 2015 Feb 20;10(2):e0117327. doi: 10.1371/journal.pone.0117327 (PMC4336289; doi:10.1371/journal.pone.0117327)
Supplement: S6 Table — (DOCX) [file pone.0117327.s006.docx]

**Table S6 Primers used to reverse transcription and quantify the levels of pri-miRNAs by qRT-PCR.**

| Pri-miRNA | Primers Name | Primers Sequence (5ˊ-3ˊ) | Note |
| --- | --- | --- | --- |
| Pri-miR-133a | Pri-miR-133aqU | TTGGTCCCCTTCAACCAGCTGTAGC | Reverse Primer qRT-PCR Primers |
|  | Pri-miR-133aqL | GTGATGCTGTGGTGTGCAGCAGACA |  |
| Pri-miR-133b | Pri-miR-133bqU | CGGGATCTGATTCATGCTAATG | Reverse Primer qRT-PCR Primers |
|  | Pri-miR-133bqL | TCTCTTCTGTTTCTCCAAGGAC |  |
| Pri-let7a | Pri-let7a-qU | GGAAGGTTTGTTTCAGTTCCAC | Reverse Primer qRT-PCR Primers |
|  | Pri-let7a-qL | TACAACCTACTACCTCATCCCA |  |
| Pri-miR-27b | Pri-miR-27bqU | TGCACCTGAAGAGAAGGTGAGA | Reverse Primer qRT-PCR Primers |
|  | Pri-miR-27bqL | TCACACATCATCTACCGCTGAC |  |
| Pri-miR-29a | Pri-miR-29aqU | CAACAGGTCAATGACGCAACATCTC | Reverse Primer qRT-PCR Primers |
|  | Pri-miR-29aqL | AACACGTTCTCTCTCCTTAGTCAG |  |
| Pri-miR-128-2 | Pri-miR-128-2qU | AAGGCTAGGGAGTCATGTTAGG | Reverse Primer qRT-PCR Primers |
|  | Pri-miR-128-2qL | GTGACACAGTAGGGAAAGAGAC |  |
| GAPDH | GAPDHqU | AGAAGGCTGGGGCTCATTTG | Reverse Primer qRT-PCR Primers |
|  | GAPDHqL | AGGGGCCATCCACAGTCTTC |  |
